# Supplementary material for: Clonal architecture predicts clinical outcomes and drug sensitivity in acute myeloid leukemia
Source: Nat Commun. 2021 Dec 13;12:7244. doi: 10.1038/s41467-021-27472-5 (PMC8669028; doi:10.1038/s41467-021-27472-5)
Supplement: Supplementary file 1 — Supplementary Information [file 41467_2021_27472_MOESM1_ESM.pdf]

a

Author	PMID	n. pts in study	Genotyping assay	VAF	n. pts with outcomes
Majeti	27526324, 28729405	50	Targeted	Yes	50
TCGA	23634996	200	WES (150), WGS (50)	Yes	200
Papaemmanuil et al.	27276561	1540	Targeted (111 genes)	Yes	1540
Tyner et al.	30333627	562	WES	Yes	504
Welch et al.	27959731	116	Exome & Targeted (264)	Yes	54
Lindsley ey al.	25550361	194	Targeted (82 genes)	Yes	183
Au et al.	26796102	50	Targeted (54 genes)	No	0
Wang et al.	27062340	95	Targeted (410 genes)	No	0
Garg et al.	26438511	80	WES & targeted	No	77
Greif et al.	29330206	50	WES	Yes	0
Huet et al.	29866812	155	Targeted	No	155
Hirsch et al.	27534895	74	Targeted (122 genes)	Yes	74

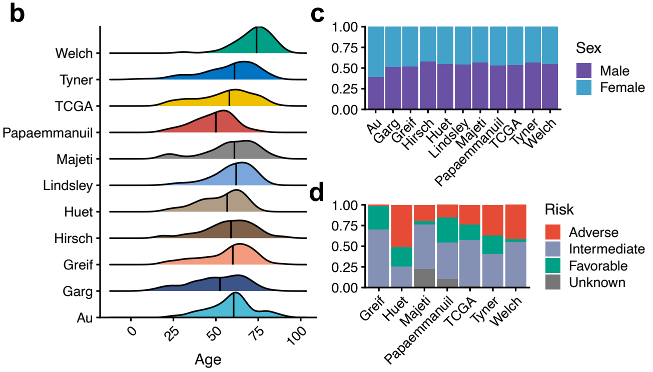

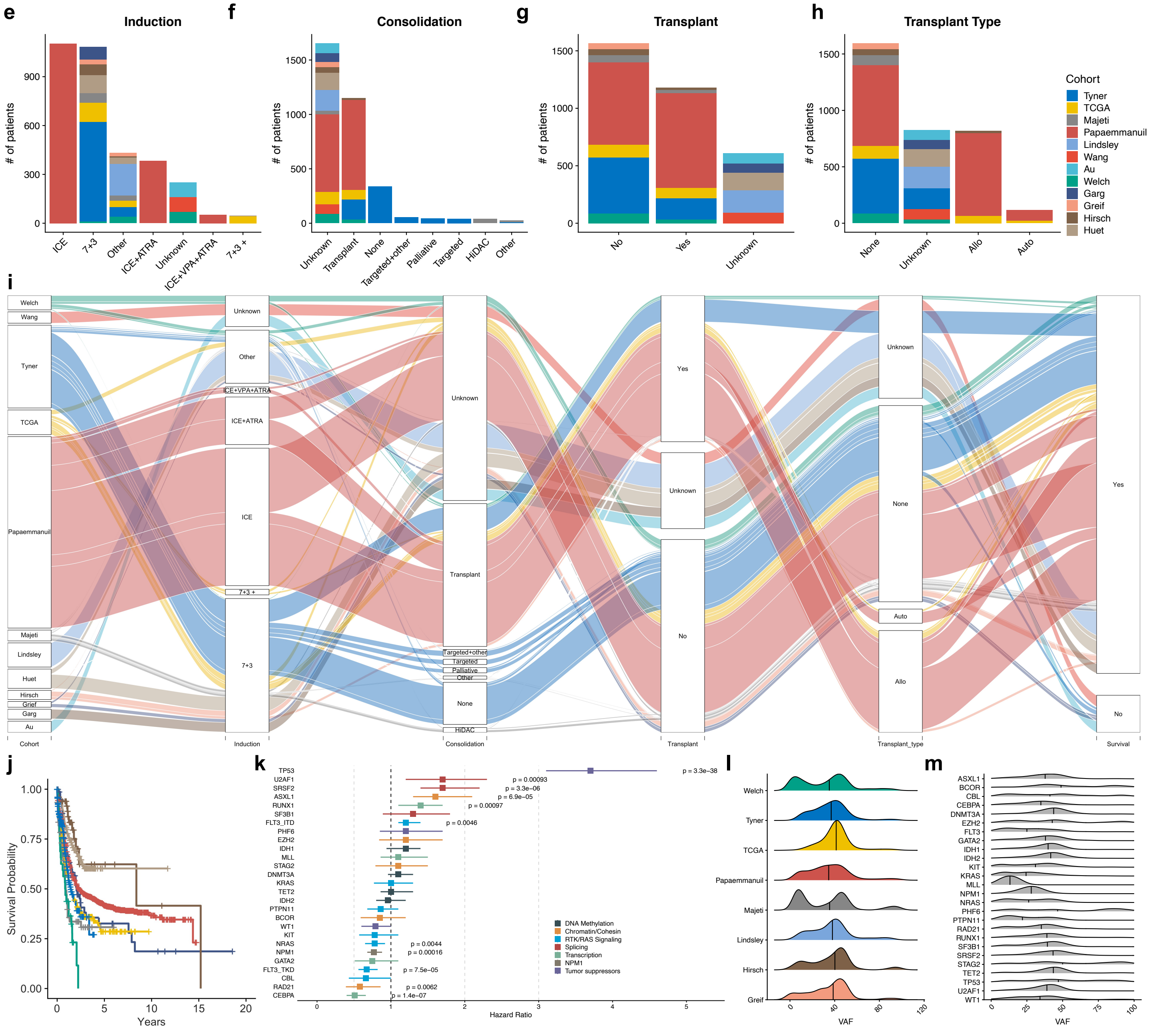

Supplementary Fig. 1: Cohort characteristics. **a** Summary table of data available per cohort. **b** Density plot for age distribution in cohorts reporting age. **c** Percentage of patients in each cohort based on reported sex. **d** Distribution of ELN 2017 risk as a normalized proportion per cohort where risk was reported. **e-h** Distribution of induction (e), consolidation (f), transplant status (g), and transplant type (h) reported in each study. **i** Alluvial plot depicting the flow of available treatment information for the studies included in our cohort. Ribbons are colored based on study and the width of each ribbon is scaled based on the number of patients with a subset of shared features. Because of the extremely heterogeneous treatment histories, patients were grouped into broad treatment bins based on manual curation. **j** Overall survival per cohort for the de novo subset of patients in our study based on Cox proportional-hazards modeling. **k** Forest plot depicting univariate Cox proportional-hazards regression results for the most frequent mutations in the de novo cohort (n = 1,857 patients). Points represent the hazard ratio between mutated and wild-type patients. Error bars represent the 95% confidence intervals of the hazard ratios. **l** Density plot for all reported VAFs per study. **m** Distribution of VAFs for the 21 most common mutations in AML.

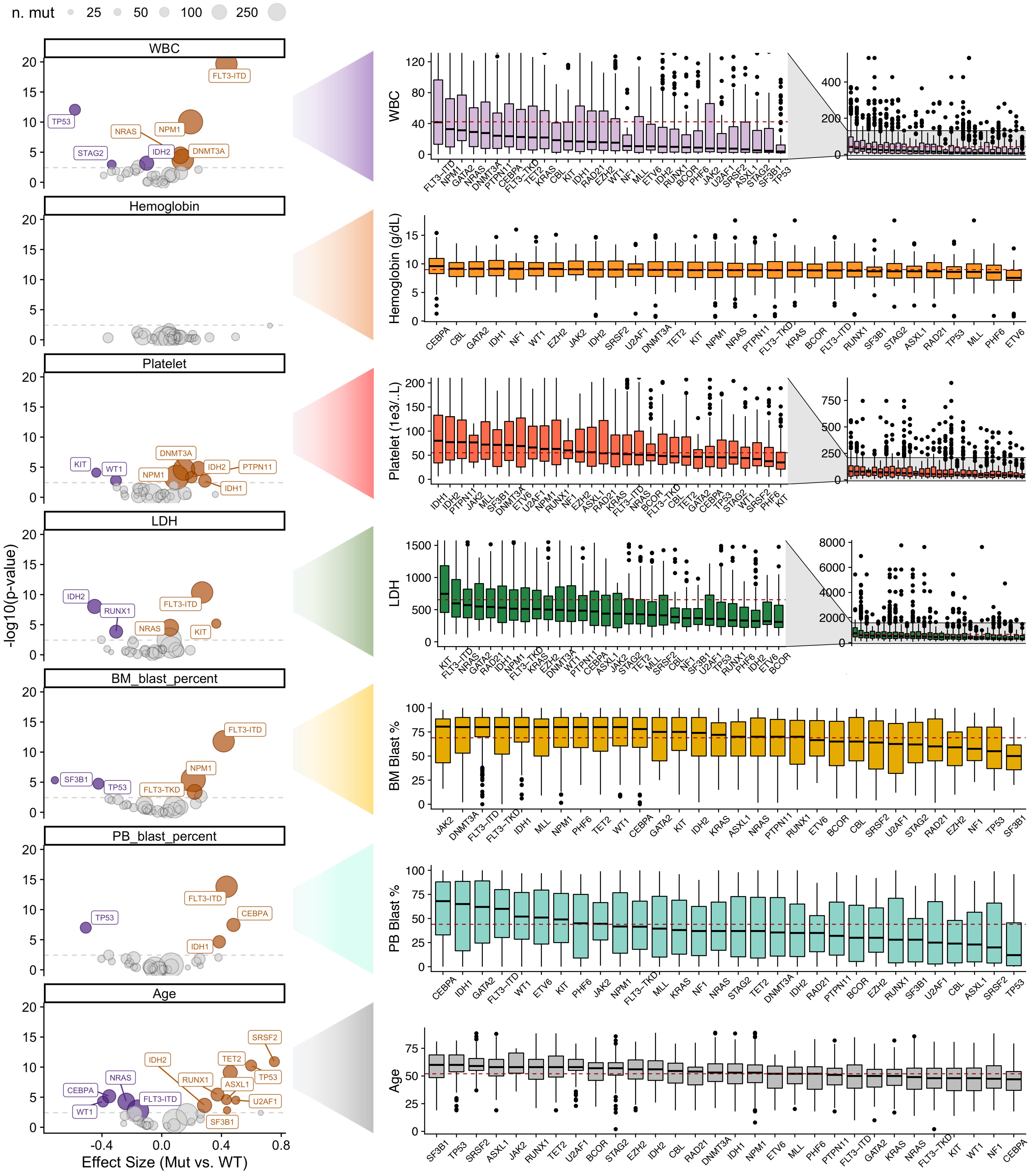

Supplementary Fig. 2: Association between individual genotypes and clinical features of AML. Statistical correlation (left) and distribution (right) between clinical features and individual genotypes for the de novo patients in our cohort. Orange points (left panels) indicate statistically significant (Bonferroni FDR < 0.05) positive effect sizes while purple points (left panels) indicate negative effect sizes. Effect sizes (Cohen's *d*) were calculated between mutated and wild-type patients. P-values were calculated using a two-sided Wilcoxon rank sum test. Dashed red lines (right panels) indicate the mean value for that feature across all mutations. For each distribution, the boxplot represents the boundaries for the first and third quartiles with a line at each median; whiskers delimit the highest data point below the third quartile +1.5x the interquartile distance and the lowest data point above the first quartile -1.5x the interquartile distance.

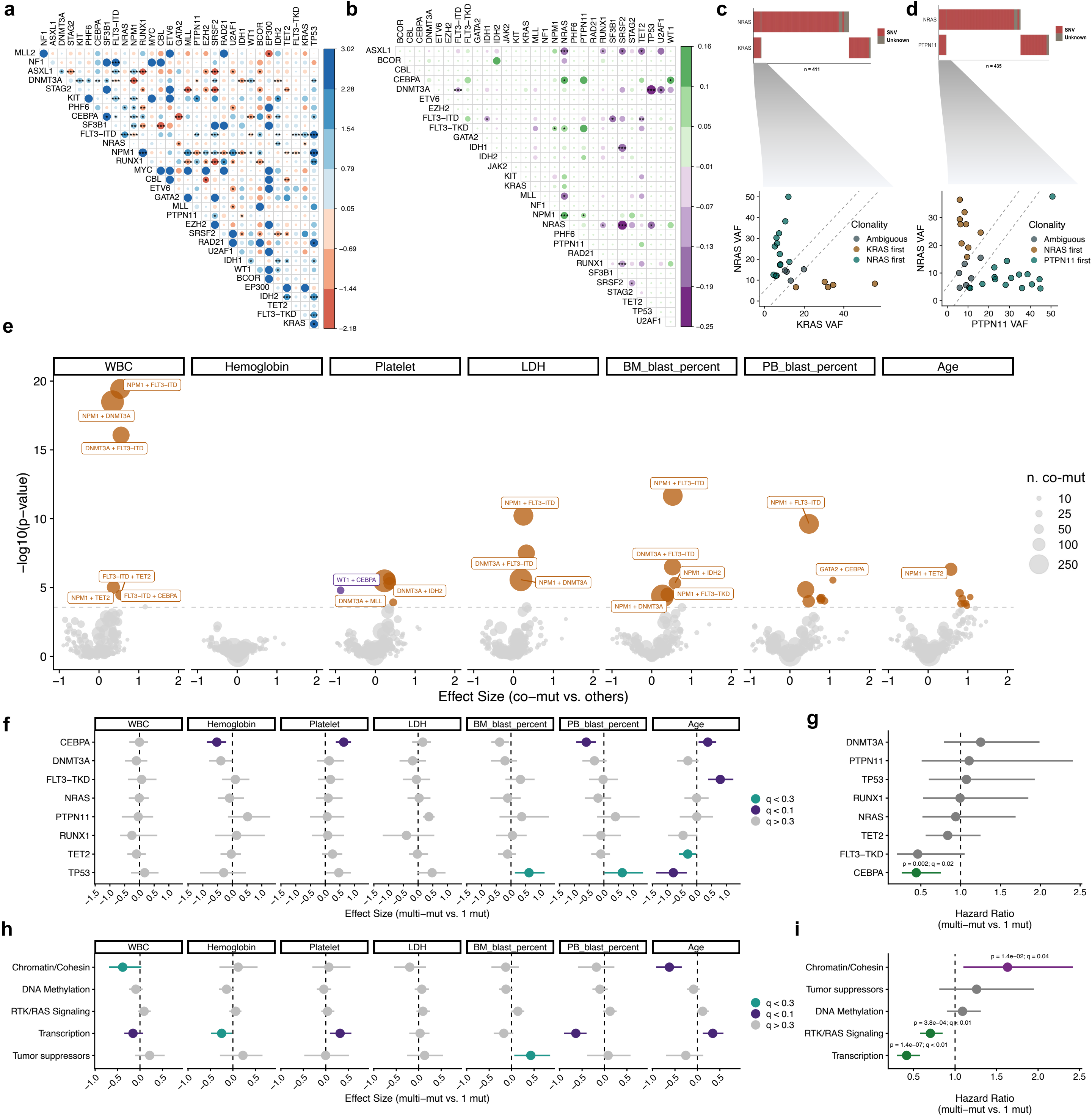

Supplementary Fig. 3: Mutation co-occurrence associates with survival outcomes and clinical features of disease presentation. **a** Co-occurrence and mutual exclusivity of the most frequent mutations present in de novo AML was performed using a two-sided Fisher's Exact test. Point size and color are based on the magnitude and directionality of the odds ratio based on co-occurrence (red) or mutual exclusivity (blue). Asterisks representing the FDR-corrected significance. **b** Correlation plot depicting the log-transformed hazard ratio compared to wild-type patients, with green depicting better prognosis (HR ≤ 1) and purple representing worse prognosis (HR ≥ 1). **c-d**) Representative examples of how binary pairwise mutation analysis can miss informative patterns of clonality. For patients with co-occurring *NRAS* and *KRAS* mutations (**c**) or *NRAS* and *PTPN11* mutations (**d**), VAF scatterplots reveals an interesting pattern of inverse clonality, indicating the presence of both major and minor clones driven by different alterations in RAS/MAPK signaling within the same patient. **e** Correlation between pairwise genotypes and clinical features of disease presentation. All genotypes with q < 0.1 are colored and annotated according to effect size (purple = negative effect size; orange = positive effect size). Effect sizes (Cohen's *d*) were calculated between co-mutated and all other patients. P-values were calculated using a two-sided Wilcoxon rank sum test. **f** Effect size of clinical features between patients with multiple mutations in the same gene compared to patients with a single mutation in that gene (n = 1,425 patients). Points represent the effect size for each clinical variable between multi-mutated and single mutated patients. Significant associations are colored based on the level of significance (Bonferroni FDR < 0.3); bars represent the 95% confidence intervals of the effect sizes. **g** Forrest plot depicting the Cox proportional-hazards regression results for overall survival between multi-mutated and single mutated patients (n = 1,047 patients). Points represent the hazard ratio for each mutation between multi-mutated and single mutated patients. Bars represent the 95% confidence intervals of the hazard ratios; green points represent better prognosis (HR ≤ 1) and purple representing worse prognosis (HR ≥ 1). **h** Effect size of clinical features between patients with multiple mutations in the same functional category compared to patients with a single mutation in that category (n = 2,036 patients). Points represent the effect size for each clinical variable between patients with multiple mutations in a functional category compared to patients with only a single mutation in that functional category. Significant associations are colored based on the level of significance (Bonferroni FDR < 0.3); bars represent the 95% confidence intervals of the effect sizes. **i** Forrest plot depicting the Cox proportional-hazards regression results for overall survival between multi-mutated and single mutated patients based on mutation category (n = 1,864 patients). Points represent the hazard ratio between patients with multiple mutations in a functional category compared to patients with only one mutation in that category. Bars represent the 95% confidence intervals of the hazard ratios; green points represent better prognosis (HR ≤ 1) and purple representing worse prognosis (HR ≥ 1). *q < 0.1; **q < 0.01, ***q < 0.001

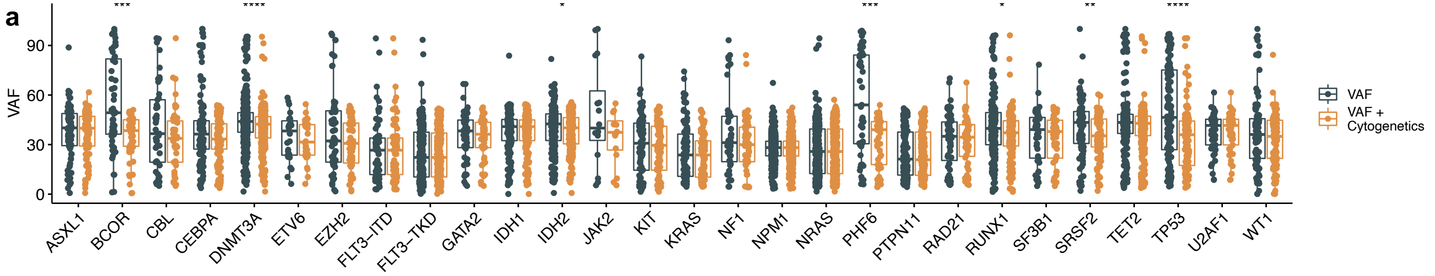

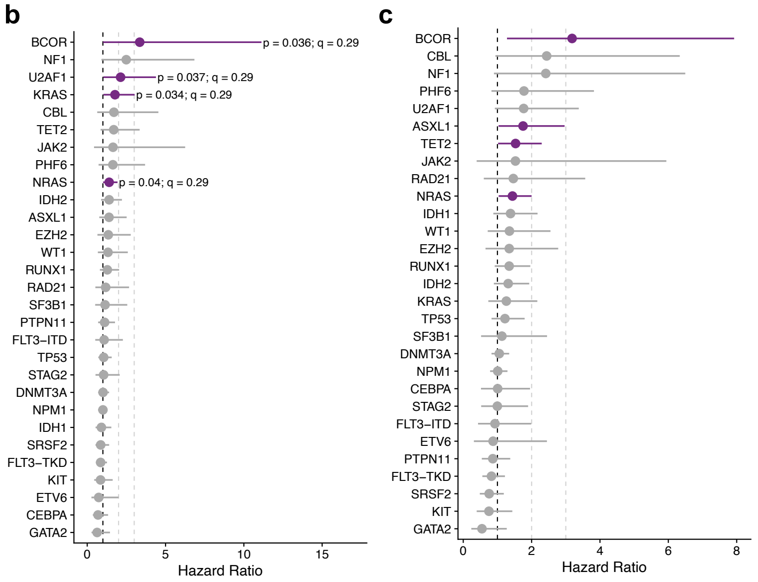

d

Gene	VAF threshold	HR	Lower CI	Upper CI	p-value	q-value
ASXL1	24.1	2.44593767	1.13	5.3	0.01980609	0.07179707
BCOR	37.74	3.94586951	1.69	9.19	0.00065775	0.01838588
CBL	34.75	2.44041962	0.94	6.33	0.05847409	0.12782773
CEBPA	18	0.4889796	0.22	1.08	0.07052564	0.12782773
DNMT3A	32.63	1.31728214	0.97	1.79	0.0762812	0.13012676
ETV6	23.47	2.29998632	0.52	10.22	0.26008085	0.29009018
EZH2	38.87	1.71341368	0.84	3.51	0.1360809	0.18758348
FLT3-ITD	17.65	0.65695363	0.3	1.44	0.29202343	0.31365479
FLT3-TKD	46.15	0.48392026	0.22	1.04	0.05835546	0.12782773
GATA2	19.16	0.22504085	0.07	0.68	0.00391878	0.03788157
IDH1	44.5	1.4035701	0.91	2.17	0.12675856	0.18544376
IDH2	33.5	1.47819776	0.99	2.22	0.05752072	0.12782773
JAK2	9.5	2.51008586	0.31	20.26	0.37195563	0.37195563
KIT	19.28	0.59423968	0.3	1.17	0.12789225	0.18544376
KRAS	29.03	1.69223435	0.98	2.94	0.05829089	0.12782773
NF1	34.38	3.67437285	1.36	9.94	0.00632152	0.045831
NPM1	34.75	1.26100524	0.88	1.81	0.20923453	0.24271205
NRAS	39.4	1.56151452	1.11	2.2	0.01056648	0.05884314
PHF6	22.92	4.99812719	1.71	14.59	0.00126799	0.01838588
PTPN11	39.61	1.85318017	1.12	3.06	0.0143665	0.05951834
RAD21	8.92	2.52870581	0.34	18.82	0.34790026	0.36032527
RUNX1	39.05	1.38724005	0.95	2.02	0.086423	0.13923706
SF3B1	46.67	2.15344405	0.93	5	0.06742429	0.12782773
SRSF2	35.29	0.73352264	0.47	1.15	0.17466937	0.21105882
STAG2	47.92	0.27923541	0.07	1.16	0.06025071	0.12782773
TET2	38.68	1.83164247	1.13	2.96	0.01217444	0.05884314
TP53	12.5	1.48483728	0.84	2.61	0.16704692	0.21062437
U2AF1	42.36	1.81752125	0.95	3.46	0.06575014	0.12782773
WT1	42.51	1.58105905	0.85	2.93	0.14230471	0.18758348

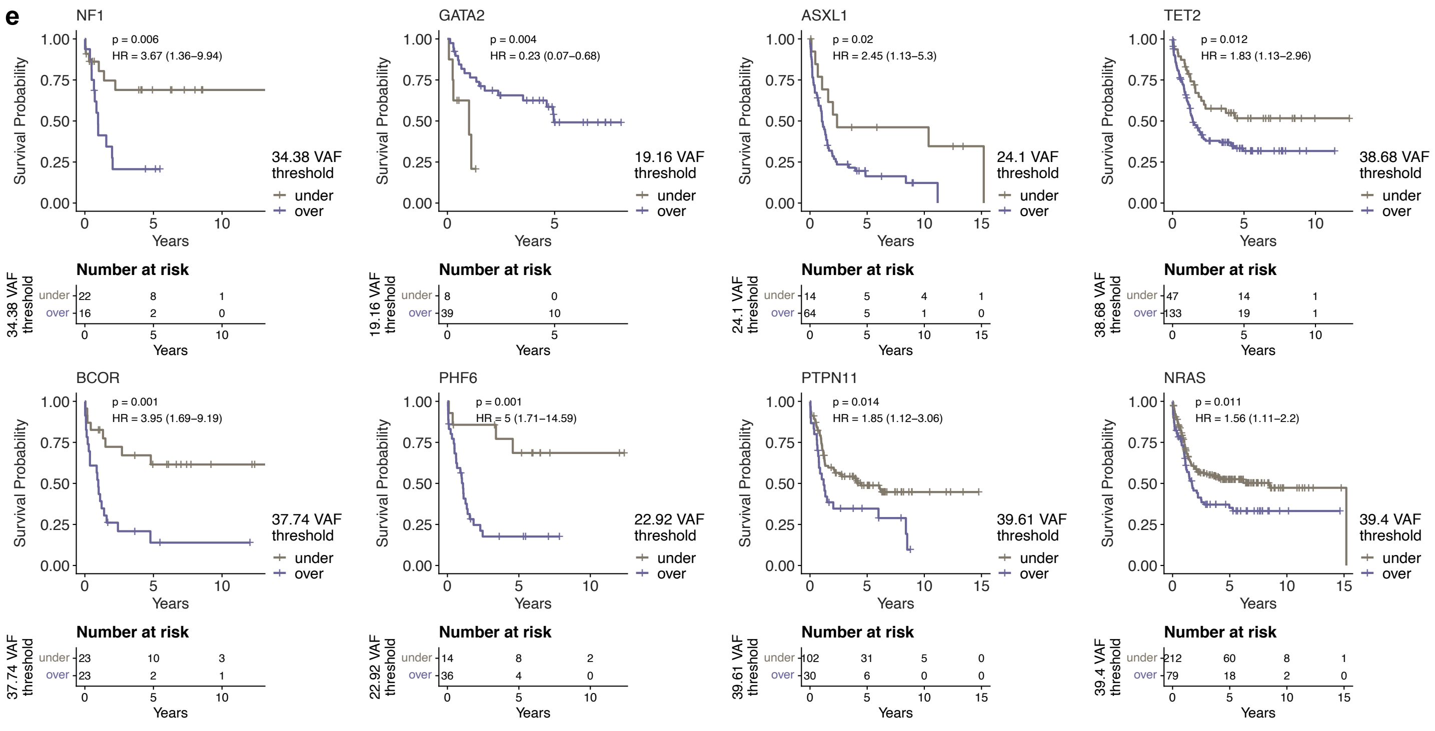

Supplementary Fig. 4: VAF correction and optimal survival thresholds. a Raw and copy number-corrected VAFs for the most frequent mutations in the de novo cohort (n = 1,642 patients). For each distribution, the boxplot represents the boundaries for the first and third quartiles with a line at each median; whiskers delimit the highest data point below the third quartile +1.5x the interquartile distance and the lowest data point above the first quartile -1.5x the interquartile distance. Differences in mean VAF distribution before and after CNA correction was tested using a two-sided Wilcoxon rank-sum test. *p ≤ 0.05; **p ≤ 0.01; ***p ≤ 0.001; ****p ≤ 0.001. **b** Forest plot summarizing univariate Cox proportional-hazards regression modeling of recurrent mutations based on static VAF level of 30% in the de novo cohort (n = 1,587 patients). Bars represent the 95% confidence intervals of the hazard ratios. **c** Forest plot summarizing univariate Cox proportional-hazards regression modeling of recurrent mutations based on median VAF thresholds per gene in the de novo cohort (n = 1,587 patients). Bars represent the 95% confidence intervals of the hazard ratios. **d** Summary table of optimal VAF thresholds for prognostic stratification based on maximally selected rank statistics. Hazard ratios and confidence intervals were calculated using standard Cox proportional-hazards regression; q-values were calculated in terms of the false discovery rate using Bonferroni correction. **e** Kaplan-Meier plots for mutations showing significant risk stratification based on dynamic VAF thresholds. Hazard ratios and confidence intervals were calculated using standard Cox proportional-hazards regression; p-values were calculated using a two-sided log-rank test.

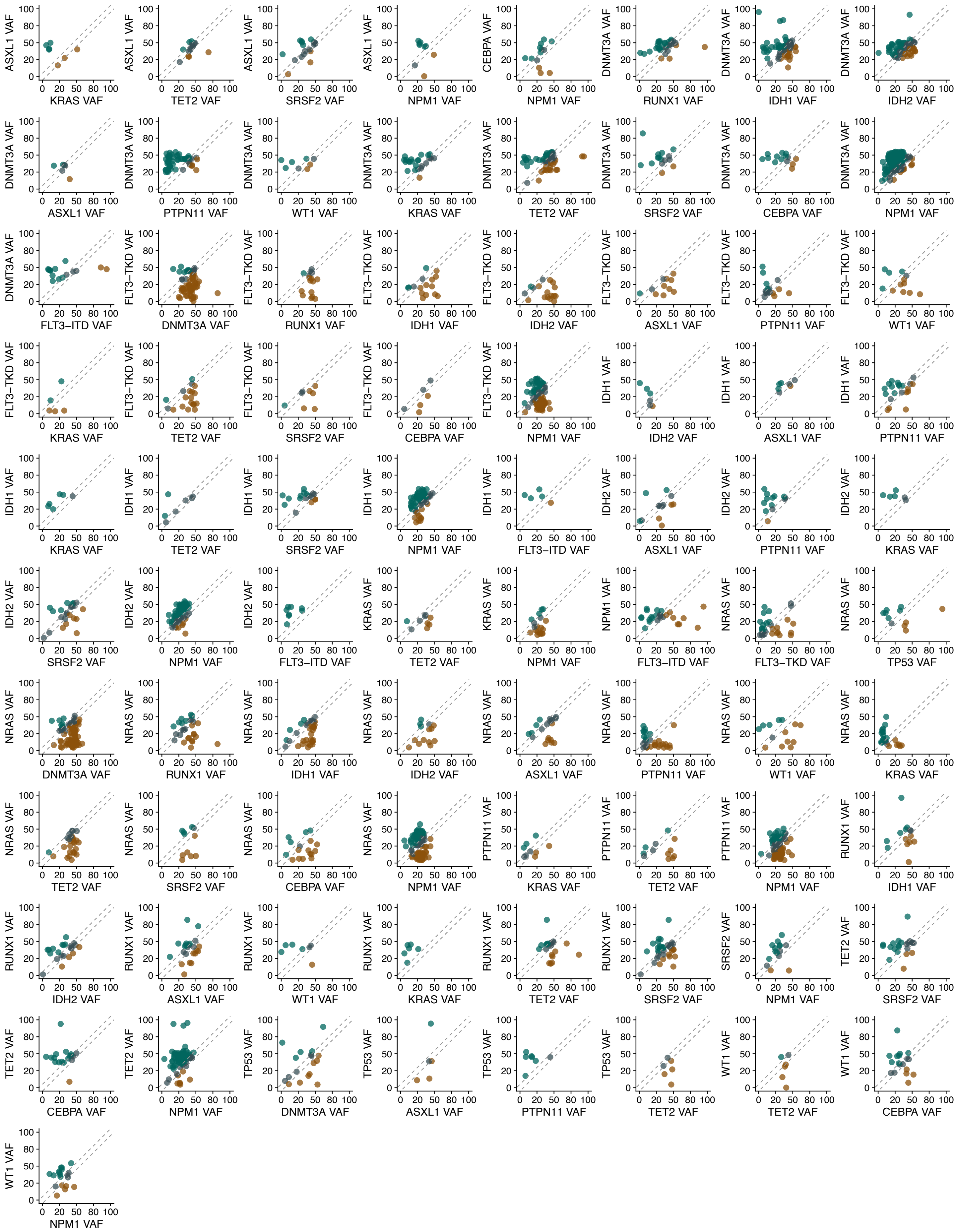

Supplementary Fig. 5: Pairwise VAF relationships in de novo AML. Copy number-corrected VAF scatterplots for all pairwise genotypes where patients show co-occurring mutations (n co-occur ≥ 5). Dotted lines represent the threshold for ambiguous ordering.

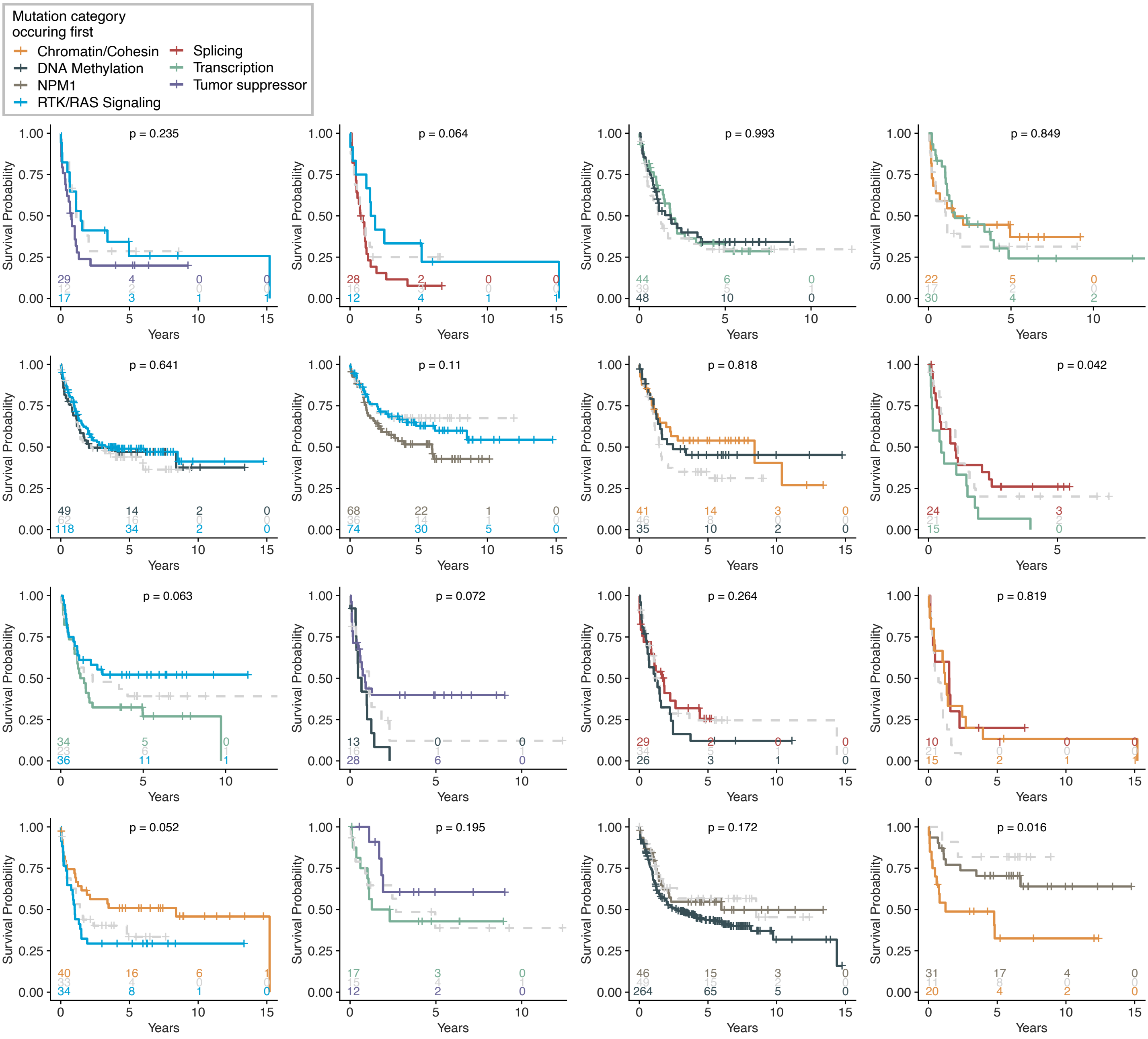

Supplementary Fig. 6: Correlation between survival and the order of acquisition for different mutation categor. Kaplan-Meier plots for patient survival based on the inferred ordering of functional mutation categories based on univariate Cox proportional-hazards regression analysis. Grey curves represent ambiguous ordering. Reported p-values are calculated between functional categories with assigned ordering.

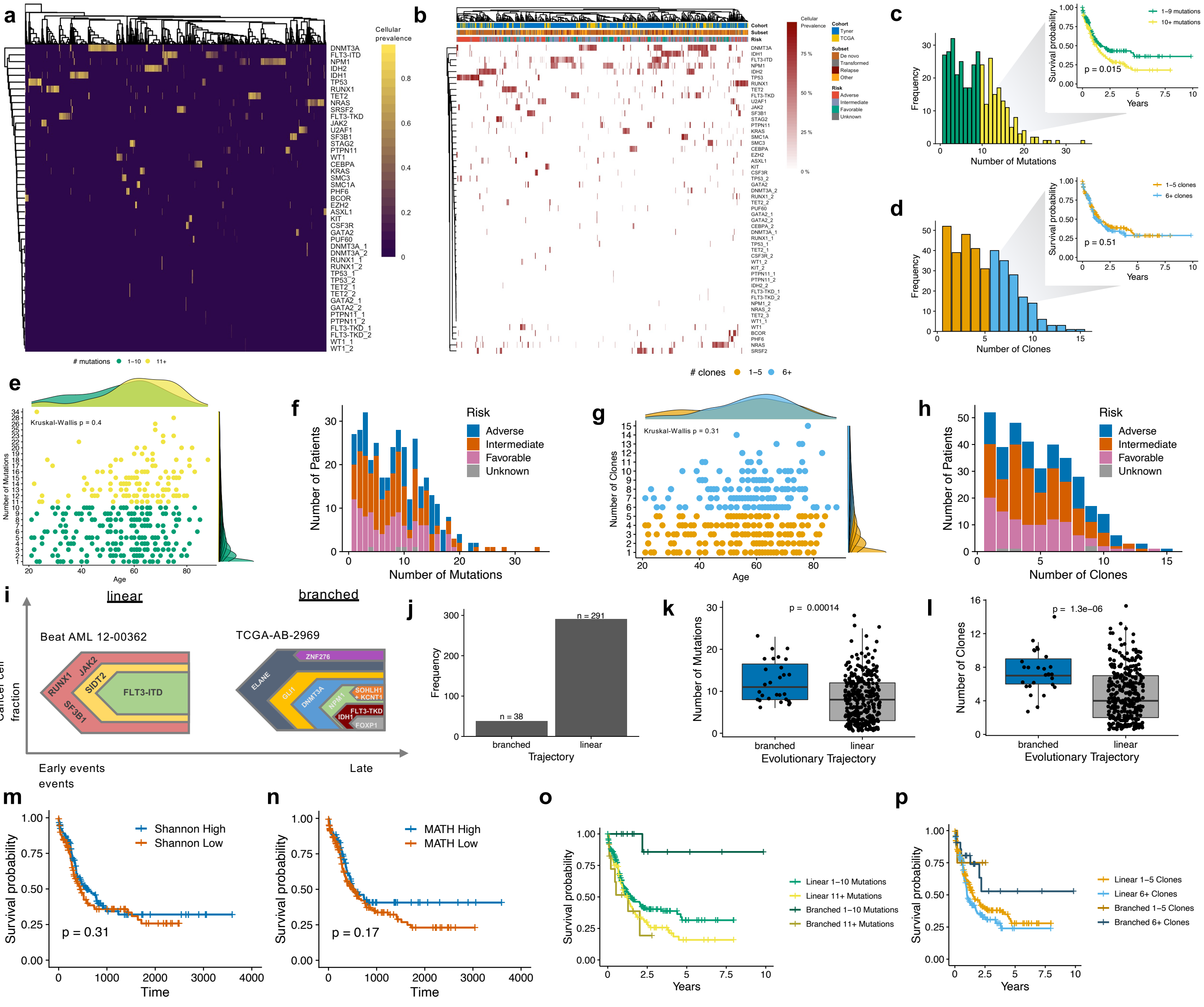

Supplementary Fig. 7: Features of clonality in de novo AML. a Heatmap of PyClone results for cellular prevalences of recurrent mutations clustered by unique clonal populations. Each column is a distinct clonal population predicted by PyClone. For clones with multiple mutations in the same gene, mutations are assigns based on their clonal dominance of cancer cell fraction (CCF) (e.g. DNMT3A_1 CCF > DNMT3A_2 CCF). **b** Heatmap of PyClone results for cellular prevalences of recurrent mutations clustered by patient samples. **c** Histogram and survival based on median protein-coding mutation burden per patient in de novo samples. p-value for the difference in survival strata was calculated using a two-sided log-rank test. **d** Histogram and survival based on median clonal abundance per patient in de novo samples. P-value for the difference in survival strata was calculated using a two-sided log-rank test. **e** Correlation between age and mutation burden. A two-sided Kruskal-Wallis test was used to calculate the reported p-value. **f** Histogram of mutation burden per patient stratified by ELN risk category. **g** Correlation between age and clonal burden. A two-sided Kruskal-Wallis test was used to calculate the reported p-value. **h** Histogram of clonal burden per patient stratified by ELN risk category. **i** Schematic showing example plots for predicted evolutionary architectures. **j** Bar plot of the number of assigned linear or branched architectures in de novo samples. **k, l** Boxplot showing statistically significant enrichment of higher mutational (**k**) and clonal (**l**) burden in samples with a branched architecture (n = 409 biologically independent samples). For each distribution, the boxplot represents the boundaries for the first and third quartiles with a line at each median; whiskers delimit the highest data point below the third quartile +1.5x the interquartile distance and the lowest data point above the first quartile -1.5x the interquartile distance. Reported p-values are calculated using a two-sided Welch t-test. **m-n** Kaplan-Meier plots showing no significant differences in outcomes based on Shannon diversity index (**m**) or MATH score (**n**) per patient; p-values were calculated using a two-sided log-rank test. **o, p** Kaplan-Meier plots showing significant stratification based on mutational and clonal burden within patients with branched architectures.

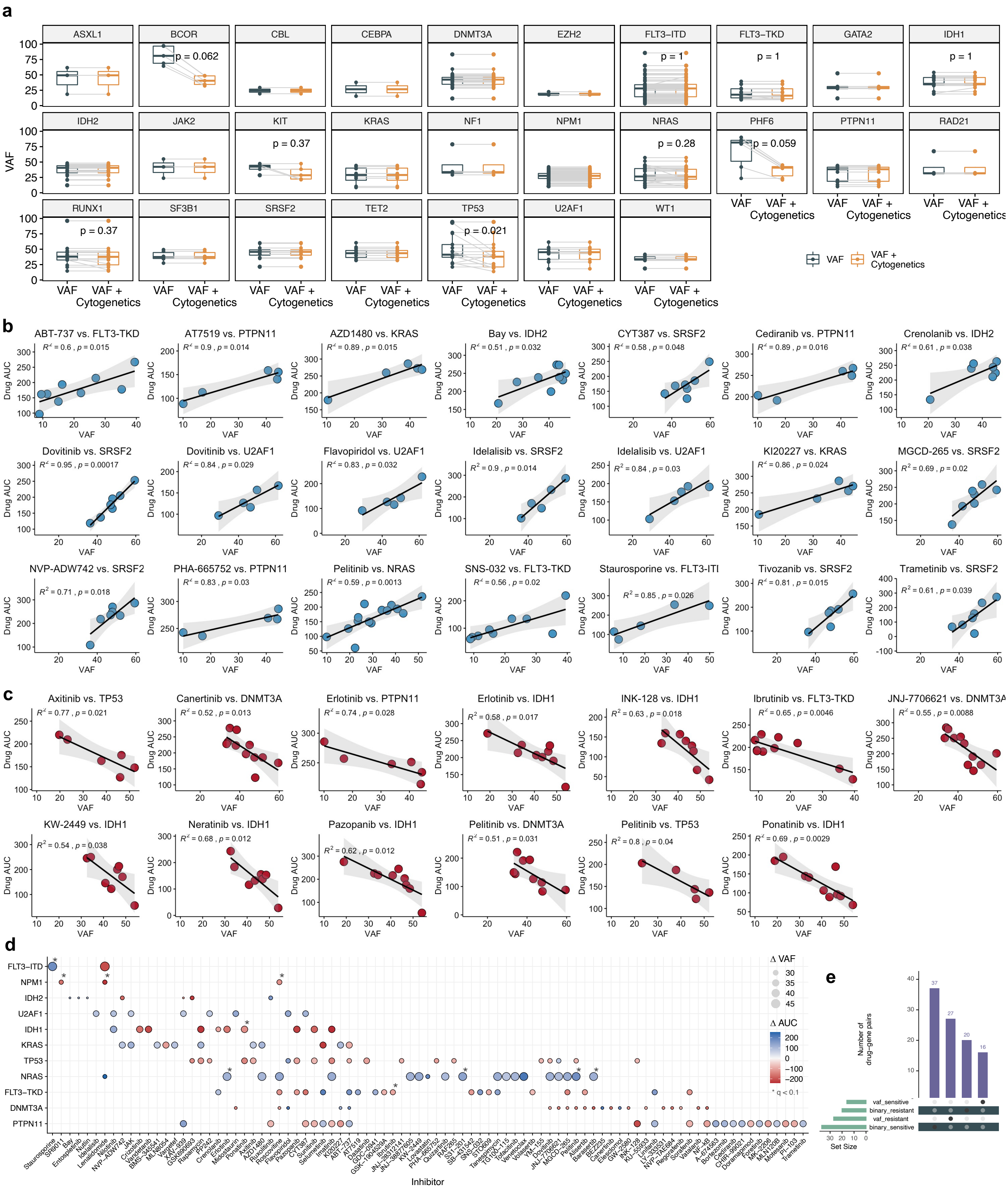

Supplementary Fig. 8: VAF correlations with drug sensitivity in de novo AML. **a** Raw and copy number-corrected VAFs for the most frequent mutations in the de novo cohort with total drug screening data (n = 202 biologically independent samples). For each distribution, the boxplot represents the boundaries for the first and third quartiles with a line at each median; whiskers delimit the highest data point below the third quartile +1.5x the interquartile distance and the lowest data point above the first quartile -1.5x the interquartile distance. Differences in mean VAF distribution before and after CNA correction was tested using a two-sided Wilcoxon rank-sum test. **b, c** Scatterplots for resistant (**b**) and sensitive (**c**) trends observed from linear regression of AUC against VAF for the most significant ($p \leq 0.05$; $R^2 \geq 0.5$) drug-gene pairs. For each scatterplot, shaded bands represent 95% confidence intervals for each linear regression. For each error band, the measure of center is the line of best fit as derived from linear regression between the drug AUC and VAF for each mutation-drug pair. **d** Dotplot for all drug-gene correlations identified through linear regression of drug AUC against mutation VAF in de novo AML samples. Points are sized based on the range of VAFs for each mutation and colored based on the type of drug sensitivity trend (red – sensitive; blue – resistant). * FDR ≤ 0.1. **e** UpSet plot showing the lack of overlap for significant drug-gene relationships observed from categorical (mutated vs. wild-type) analysis compared to continuous (VAF) analysis.

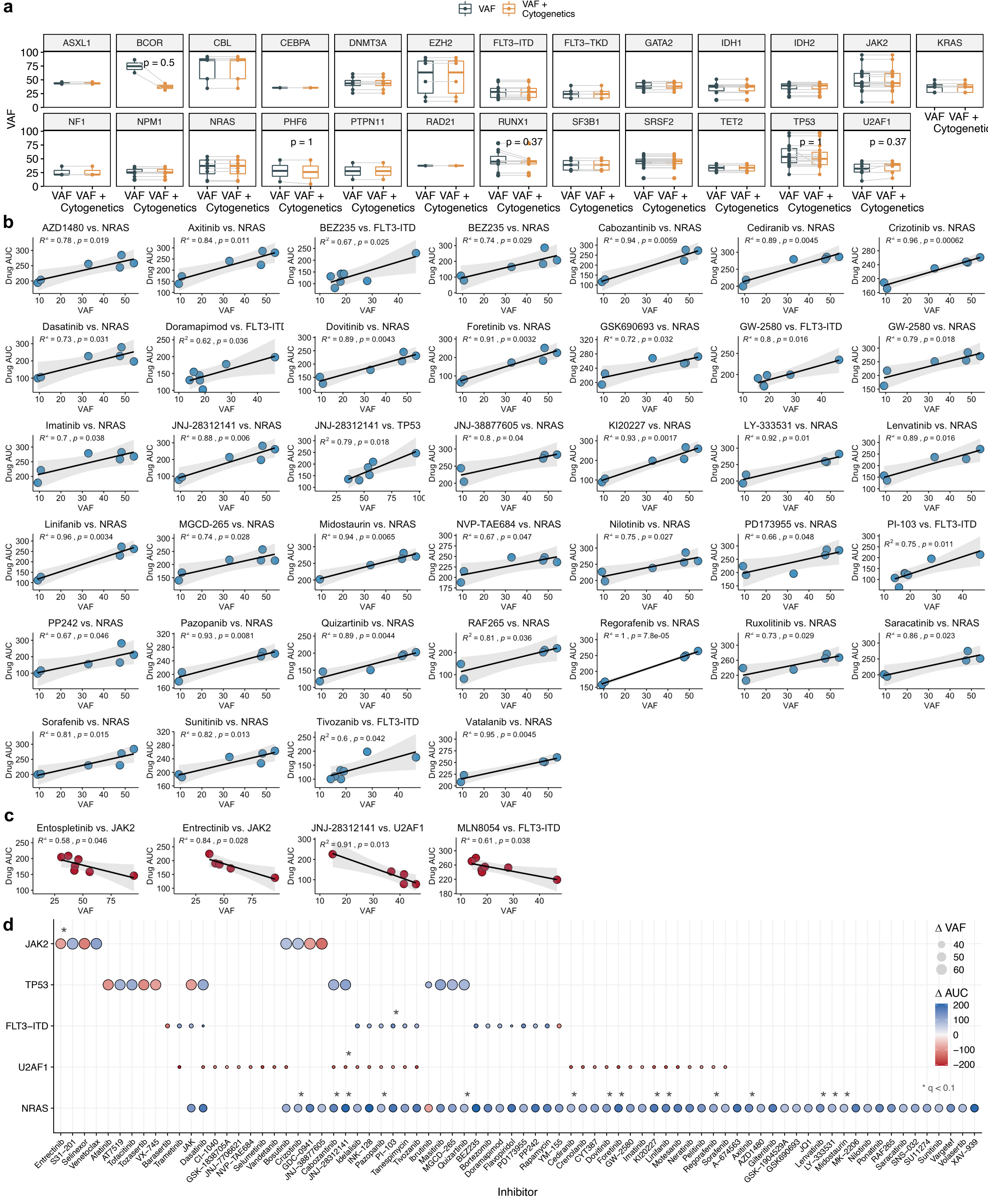

Supplementary Fig. 9: VAF correlations with drug sensitivity in secondary AML. **a** Raw and copy number-corrected VAFs for the most frequent mutations with total drug screening data in the secondary AML samples (n = 88 biologically independent samples). For each distribution, the boxplot represents the boundaries for the first and third quartiles with a line at each median; whiskers delimit the highest data point below the third quartile +1.5x the interquartile distance and the lowest data point above the first quartile -1.5x the interquartile distance. Differences in mean VAF distribution before and after CNA correction was tested using a two-sided Wilcoxon rank-sum test. **b, c** Scatterplots for resistant (**b**) and sensitive (**c**) trends observed from linear regression of AUC against VAF for significant ($p \leq 0.05$; $R^2 \geq 0.5$) drug-gene pairs. For each scatterplot, shaded bands represent 95% confidence intervals for each linear regression. For each error band, the measure of center is the line of best fit as derived from linear regression between the drug AUC and VAF for each mutation-drug pair. **d** Dotplot for all drug-gene correlations identified through linear regression of drug AUC against mutation VAF in de novo AML samples. Points are sized based on the range of VAFs for each mutation and colored based on the type of drug sensitivity trend (red – sensitive; blue – resistant). * FDR ≤ 0.1.
