# Supplementary material for: Clonal architecture predicts clinical outcomes and drug sensitivity in acute myeloid leukemia
Source: Nat Commun. 2021 Dec 13;12:7244. doi: 10.1038/s41467-021-27472-5 (PMC8669028; doi:10.1038/s41467-021-27472-5)
Supplement: Supplementary file 3 — Reporting Summary [file 41467_2021_27472_MOESM3_ESM.pdf]

nature portfolio

Corresponding author(s): Ravindra Majeti

Last updated by author(s): 2021/11/15

Reporting Summary

Nature Portfolio wishes to improve the reproducibility of the work that we publish. This form provides structure for consistency and transparency in reporting. For further information on Nature Portfolio policies, see our Editorial Policies and the Editorial Policy Checklist.

Statistics

For all statistical analyses, confirm that the following items are present in the figure legend, table legend, main text, or Methods section.

n/a	Confirmed	
☐	☒	The exact sample size (*n*) for each experimental group/condition, given as a discrete number and unit of measurement
☐	☒	A statement on whether measurements were taken from distinct samples or whether the same sample was measured repeatedly
☐	☒	The statistical test(s) used AND whether they are one- or two-sided<br>*Only common tests should be described solely by name; describe more complex techniques in the Methods section.*
☐	☒	A description of all covariates tested
☐	☒	A description of any assumptions or corrections, such as tests of normality and adjustment for multiple comparisons
☐	☒	A full description of the statistical parameters including central tendency (e.g. means) or other basic estimates (e.g. regression coefficient) AND variation (e.g. standard deviation) or associated estimates of uncertainty (e.g. confidence intervals)
☐	☒	For null hypothesis testing, the test statistic (e.g. *F*, *t*, *r*) with confidence intervals, effect sizes, degrees of freedom and *P* value noted<br>*Give P values as exact values whenever suitable.*
☒	☐	For Bayesian analysis, information on the choice of priors and Markov chain Monte Carlo settings
☒	☐	For hierarchical and complex designs, identification of the appropriate level for tests and full reporting of outcomes
☐	☒	Estimates of effect sizes (e.g. Cohen's *d*, Pearson's *r*), indicating how they were calculated

Our web collection on statistics for biologists contains articles on many of the points above.

Software and code

Policy information about availability of computer code

Data collection	All code used to download and process data for this project was written in R version 4.0.4 (2021-02-05) and the RStudio statistical software platform v1.2.1114. Code for this project has been deposited at https://github.com/brooksbenard/Meta_AML
Data analysis	All code used in the data analysis for this project was written in R version 4.0.4 (2021-02-05) and the RStudio statistical software platform v1.2.1114. Code for this project has been deposited at https://github.com/brooksbenard/Meta_AML. Relevant packages used in the data analysis are: PyClone (v0.13.0; Python v2.7); ClonEvol (clonevol_0.99.11); vegan_2.5-7; BradleyTerry2_1.1-2; stats_4.0.4; survminer_0.4.9; MaxStat v. 0.7-25; effsize_0.8.1

For manuscripts utilizing custom algorithms or software that are central to the research but not yet described in published literature, software must be made available to editors and reviewers. We strongly encourage code deposition in a community repository (e.g. GitHub). See the Nature Portfolio guidelines for submitting code & software for further information.

Data

Policy information about availability of data

All manuscripts must include a data availability statement. This statement should provide the following information, where applicable:

- Accession codes, unique identifiers, or web links for publicly available datasets
- A description of any restrictions on data availability
- For clinical datasets or third party data, please ensure that the statement adheres to our policy

Processes mutation calls and clinical data for the publicly available datasets used in this study were obtained from the following links: TCGA (http://download.cbioportal.org/laml_tcga_pub.tar.gz); Tyner (https://static-content.springer.com/esm/art%3A10.1038%2Fs41586-018-0623-z/MediaObjects/41586_2018_623_MOESM3_ESM.xlsx); Papaemmanuil (https://github.com/gerstung-lab/AML-multistage/blob/master/data/

AMLSG_Clinical_Anon.RData?raw=true; https://raw.githubusercontent.com/gerstung-lab/AML-multistage/master/data/AMLSG_Genetic.txt); Lindsley (https://ashpublications.org/blood/article/125/9/1367/34220/Acute-myeloid-leukemia-ontogeny-is-defined-by); Wang (https://www.oncotarget.com/article/7028/); Au (https://diagnosticpathology.biomedcentral.com/articles/10.1186/s13000-016-0456-8); Welch (https://www.nejm.org/doi/full/10.1056/NEJMoa1605949); Garg (https://ashpublications.org/blood/article/126/22/2491/34632/Profiling-of-somatic-mutations-in-acute-myeloid); Greif (https://clincancerres.aacrjournals.org/content/early/2018/01/12/1078-0432.CCR-17-2344.figures-only?versioned=true); Hirsch (https://static-content.springer.com/esm/art%3A10.1038%2Fncomms12475/MediaObjects/41467_2016_BFncomms12475_MOESM1147_ESM.pdf; https://static-content.springer.com/esm/art%3A10.1038%2Fncomms12475/MediaObjects/41467_2016_BFncomms12475_MOESM1148_ESM.xlsx); Huet (https://ash.silverchair-cdn.com/ash/content_public/journal/blood/132/8/10.1182_blood-2018-03-840348/4/blood840348-sup-tables2.xlsx?Expires=1579153943&Signature=VCqH4IQOtZ~x9YPe1dJSz1AHegirMKv6N4euRcbvd3gsayA~RULg17B6mjZY0lrUzbbbj5D7MdLJNzI0sUk4QFDz6IKvvWsV~l1CQL4J1lOHejvckLQzXikTy-4SD62jwmDMgSeBMhXqVo6pmdHXI0iO7b8ARJV8EDZ9Bt8h~h6nIXOaf5JZqe8hehacxWIMjCWRd5OulQjpqQu10MLfK6GrpISgRGDYe1WODtkPIQ~o6qQj~eqTFGvzwbSmhWuRGSAFKEFzPzgYLiKmkQ8errjq9vI2uHgpurlwtB98gQOnizt9goXVoDPsGd0P~fk8CPPEGJdUtSkOB41b8p3JTg__&Key-Pair-Id=APKAIE5G5CRDK6RD3PGA); Majeti (https://static-content.springer.com/esm/art%3A10.1038%2Fng.3646/MediaObjects/41588_2016_BFng3646_MOESM43_ESM.xlsx; https://cancerdiscovery.aacrjournals.org/highwire/filestream/41655/field_highwire_adjunct_files/6/181878_2_supp_4135424_zsdnjx.xlsx). Raw TCGA sequencing data used in this study are available at the NIH Genomic Data Commons [https://gdc.cancer.gov]. Raw sequencing data for the Papaemmanuil study is deposited at the European Genome-Phenome Archive with accession number EGA00001000275 [https://www.ebi.ac.uk/ega]. For the Tyner study, sequencing data and clinical annotations can be found at dbGaP and the Genomic Data Commons. The dbGaP study ID is 30641 and accession ID is phs001657.v1.p1 [https://www.ncbi.nlm.nih.gov/projects/gap/cgi-bin/study.cgi?study_id=phs001657.v1.p1]. For the Welch dataset, exome sequencing data are deposited in the database of Genotypes and Phenotypes under the accession number phs000159 [https://www.ncbi.nlm.nih.gov/projects/gap/cgi-bin/study.cgi?study_id=phs000159.v11.p5]. For the Huet study, raw exome and targeted sequencing data are deposited at the European Genome-phenome Archive (EGA, https://ega-archive.org) under accession number EGAS00001001779 [https://ega-archive.org/studies/EGAS00001001779] - data are available upon request from the Data Access Committee of the MyPAC clinical research group. The aggregated mutational and clinical data frames used for the analyses in this paper are available at https://github.com/brooksbenard/Meta_AML. Source data are provided with this paper.

Field-specific reporting

Please select the one below that is the best fit for your research. If you are not sure, read the appropriate sections before making your selection.

☒ Life sciences ☐ Behavioural & social sciences ☐ Ecological, evolutionary & environmental sciences

For a reference copy of the document with all sections, see nature.com/documents/nr-reporting-summary-flat.pdf

Life sciences study design

All studies must disclose on these points even when the disclosure is negative.

Sample size	No statistical methods were used to predetermine sample size. For all survival analyses, we required at least 5 patients per strata as this provides improved resolution to analyze low frequency genotypes and affords reasonable statistical confidence for outcomes comparison. For statistical tests of differences in data distribution of clinical features, we required at least 10 data points per condition. For drug sensitivity analyses, we required paired drug-mutation data for at least 5 samples when performing linear regression and t-tests for differences in distributions.
Data exclusions	All pediatric, MDS, MPN, and secondary AML patients were excluded from the analyses due to major differences in clinical and molecular disease presentation.
Replication	In order to facilitate reproducibility, all raw data, results, and analysis code has been deposited at https://github.com/brooksbenard/Meta_AML
Randomization	Samples/participants were not randomized in this retrospective study. This is not relevant to the current study because we were 1) not assigning patients/animals to treatment arms and 2) specifically interested in comparing features between defined patient genotype groups.
Blinding	The investigators were not blinded to allocation during analyses and outcomes assessment. This is not relevant to the current study because we performed a retrospective observational study.

Reporting for specific materials, systems and methods

We require information from authors about some types of materials, experimental systems and methods used in many studies. Here, indicate whether each material, system or method listed is relevant to your study. If you are not sure if a list item applies to your research, read the appropriate section before selecting a response.

Materials & experimental systems

n/a	Involved in the study
☒	☐ Antibodies
☒	☐ Eukaryotic cell lines
☒	☐ Palaeontology and archaeology
☒	☐ Animals and other organisms
☒	☐ Human research participants
☒	☐ Clinical data
☒	☐ Dual use research of concern

Methods

n/a	Involved in the study
☒	☐ ChIP-seq
☒	☐ Flow cytometry
☒	☐ MRI-based neuroimaging
